# Evaluating the impact of eHealth interventions on adolescents with diabetes: a systematic review and meta-analysis

**DOI:** 10.3389/fcdhc.2025.1659146

**Published:** 2025-12-10

**Authors:** Silvia Spaggiari, Giulia Bassi, Silvia Salcuni, Daniela Di Riso

**Affiliations:** Department of Developmental Psychology and Socialization, University of Padova, Padova, Italy

**Keywords:** adolescents, diabetes mellitus, quality of life, HbA1c, eHealth, meta-analysis

## Abstract

**Background:**

Adolescence is marked by significant changes. The presence of type 1 and type 2 diabetes mellitus (T1D and T2D) amplifies these challenges, with diabetes being the second most common chronic disease among adolescents worldwide. Adolescents with diabetes are at heightened risk for mental health issues, which escalate the risk of complications. eHealth interventions using Information and Communication Technologies show promise in improving diabetes management and psychological well-being. However, research has predominantly focused on adults, leaving gaps in understanding the efficacy of these interventions for adolescents. Medical management often prioritizes physical health, neglecting psychosocial aspects.

**Objective:**

This meta-analysis aims to provide evidence on eHealth interventions’ efficacy in supporting the psychosocial well-being of adolescents with T1D and T2D, and to investigate their impact on Hemoglobin A1c (HbA1c), quality of life, diabetes distress, anxiety, and depression symptoms.

**Method:**

A PRISMA-guided systematic search was conducted. Randomized Controlled Trials (RCTs) regarding eHealth interventions for adolescents with diabetes were included. Data were pooled using Standard Mean Difference (SMD). Outcomes were quality of life and HbA1c. Intervention acceptability was assessed using the Odds Ratio (OR) of dropout rates.

**Results:**

A total of ten RCTs involving only adolescents with T1D (aged 10-22) were included in the analysis. The interventions resulted in significant improvements in quality of life (SMD = 0.73; 95% CI [0.08, 1.38]; k = 6), indicating a moderate positive effect, as well as in satisfaction with life, a subscale of the overall quality of life (SMD = 0.51, 95% CI [0.08, 0.95]; k = 3). For HbA1c levels, however, the effect was small and not statistically significant (SMD = -0.21; 95% CI [-0.69, 0.27]; k = 8). Additionally, the interventions were well accepted, as suggested by the OR of 0.47 (95% CI [-0.07, 1.01]; k = 7), indicating no significant difference in dropout rates between intervention and control groups.

**Conclusion:**

These results underscore the potential of eHealth interventions to enhance the quality of life and satisfaction with life in adolescents with T1D. Future research should continue to explore and refine eHealth interventions, ensuring an integrated approach that addresses both the medical and psychosocial needs of adolescents with diabetes.

**Systematic Review Registration:**

https://www.crd.york.ac.uk/prospero/, identifier CRD42021218623.

## 1 Introduction

Adolescence is a critical developmental phase bridging childhood and adulthood, and it is characterized by significant biological, psychological, emotional, and social transformations (1). The presence of a chronic condition, such as diabetes during this period, can pose an additional challenge for adolescents, who are already navigating the multiple changes typical of puberty (2).

Diabetes is a chronic metabolic condition, where type 1 and type 2 diabetes (T1D, T2D) are the most prevalent ones. T1D is particularly prevalent during adolescence, making it the second most widespread chronic disease among individuals in this age group (1). Both types of diabetes are associated with psychological challenges, specifically linked to the daily management of the condition and the need to find a balance between physical, affective well-being and quality of life (Babbott et al., 2023; Chaves et al., 2020). Evidence suggests a bidirectional relationship between psychological and physical effects. Recent studies have demonstrated a significant association between depressive symptoms and poorer metabolic control in adolescents with T1D. For example, a recent study found that both depression and diabetes distress are linked to increased HbA1c levels in this population (1). The research indicated that diabetes distress had a stronger correlation with HbA1c than depression, emphasizing the importance of addressing both psychological factors in diabetes management across different age groups (2). According to one study (N = 150), one third (34.7%) of adolescents and young adults with T1D screened positive for either depression (11.3%), anxiety (21.3%) or disordered eating (20.7%) (3). A study of the mental health of adolescents and young adults with T2D (N = 64) reported high depressive symptoms in 22%. Moreover, high diabetes distress, which refers to the emotional burden of living with diabetes and the relentless daily self-management, was reported by 23% of participants. When diabetes and mental health problems coexist, the risk of short- and long-term complications increases considerably (4).

In recent years, electronic health (eHealth) interventions, which applied digital technologies and electronic communication, have shown considerable potential for improving diabetes management through remote and personalized healthcare support. Various systematic reviews and meta-analyses have examined the medical and physiological outcomes of these interventions in adolescents with diabetes, finding significant improvements in glycemic control and reductions in HbA1c levels. For instance, Dougherty et al. (5) demonstrated that telemedicine interventions helped lower HbA1c in adolescents with T1D, while Lee et al. (6) found similar benefits across age groups in both T1D and T2D. More recent meta-analyses, such as those by (7) and (8), reinforced these findings, highlighting the consistent impact of eHealth on metabolic control and adherence to diabetes management.

However, despite these promising findings in medical outcomes, fewer studies have examined the efficacy of these digital interventions addressing psychosocial aspects among adolescents. A meta-analysis including 26 studies of mobile Health (mHealth) interventions for individuals with T1D across age groups found significant improvements in glycemic control and psychological indicators, such as higher life satisfaction, reduced diabetes-related worry, and better overall mental health. Notably, effects were evident in both adults and youth, though subgroup analyses suggested slightly smaller effects among younger participants, and benefits were greater when interventions included professional feedback features (9). Complementing these findings, a systematic review of 23 Randomized Control Trials (RCTs) focused specifically on children and adolescents with T1D reported that internet- and phone-based interventions significantly improved self-efficacy, although no significant effects were observed for quality of life or adherence outcomes (10). Taking together, these results suggest that while eHealth interventions hold promise for supporting adolescents’ psychological adjustment, the evidence remains mixed for broader domains such as quality of life. This underscores the need for targeted research on eHealth’s efficacy in supporting the broader psychosocial well-being of adolescents with diabetes, as comprehensive management ideally addresses both physical and psychosocial outcomes to improve their overall well-being.

In light of this, the present work is among the first meta-analyses to evaluate the efficacy of eHealth solutions derived from RCTs, targeting adolescents with T1D and T2D. The primary aim was to investigate the efficacy of these eHealth interventions in enhancing the overall quality of life, reducing anxiety, stress and depression symptoms, and maintaining optimal HbA1c levels.

## 2 Materials and methods

This meta-analysis was conducted in accordance with the Preferred Reporting Items for Systematic Reviews and Meta-Analyses (PRISMA) guidelines (11) and the recommendations outlined in the Cochrane Handbook for Systematic Reviews (12). The protocol for this meta-analysis received approval and was registered on PROSPERO in January 2021, under registration number CRD42021218623.

### 2.1 Eligibility criteria

Eligible studies were required to report at least one prespecified outcome, either a primary psychosocial outcome (quality of life, depression, anxiety, or diabetes-related distress) or the single secondary outcome, glycemic control (HbA1c). Specifically, studies were included in this meta-analysis if they met the following criteria: (i) RCTs comparing any eHealth intervention (e.g., telemedicine, digital games, web-based, smartphone-based interventions) with a control condition (e.g., no intervention, waiting list, treatment as usual (TAU), active conditions); (ii) adolescents aged between 10 and 19 years (13) with T1D or T2D; (iii) primary outcomes including anxiety and depression symptoms, diabetes distress, and quality of life; (iv) secondary outcomes including HbA1c levels. The exclusion criteria were studies involving individuals with any type of diabetes other than T1D or T2D, those with medical or psychological conditions different from or comorbid with T1D or T2D, and studies focusing on (pre)adolescents at risk of developing diabetes or diagnosed with prediabetes.

### 2.2 Primary and secondary outcomes

Primary outcomes assessed included mean changes in psychosocial factors. This meta-analysis evaluated these outcomes at the end of the eHealth intervention and at any follow-up assessments. Secondary outcomes focused on medical factors, specifically HbA1c levels. Additionally, two different sensitivity analyses were conducted as secondary outcomes: one assessing the efficacy of eHealth interventions on psychosocial symptoms, while the other examining improvements in glycemic control. Intervention acceptability was determined by analyzing drop-out rates.

### 2.3 Search strategy

Two authors (BV and SS) independently screened the titles and abstracts of the identified articles using a double-blind process to ensure impartiality. The literature search was conducted on five academic databases such as Web of Science, Scopus, PubMed, PsycInfo, and Cinhal, including studies published between 2000 and 2024. This time window was selected to ensure a comprehensive inclusion of relevant trials, and it is consistent with the updated PROSPERO protocol. The search strategy was developed in accordance with the registered PROSPERO protocol, and it included the following keywords combined using Boolean operators: *‘diabetes mellitus’, ‘diabetes mellitus type 1’, ‘diabetes mellitus type 2’, ‘eHealth’, ‘Mobile Health’, ‘mHealth’, ‘Telehealth’, ‘digital games’, ‘text message’, ‘short message’, ‘glycaemic’, ‘glucose’, ‘HbA1c’, ‘anxiety’, ‘stress’, ‘distress’, ‘depression’, ‘quality of life’, ‘adolescent’, ‘youth’, ‘young’, ‘randomized controlled trial’, ‘randomized’, ‘clinical’, and ‘experimental’.* The specific search string for each database is provided in **Supplementary Table S1** within the Supplementary Materials.

Each title and abstract were reviewed to determine if the study met the predefined inclusion criteria. Articles deemed potentially eligible based on their titles and abstracts were then subjected to a thorough full-text review by the same two authors, again using a double-blind process. This involved a thorough examination of the study’s methodology, participant characteristics, interventions, and outcomes.

If any disagreements arose regarding the inclusion of titles, abstracts, or full-text articles, the authors engaged in detailed discussions to resolve the differences. In cases where consensus could not be reached through discussion, a third author (GB) was consulted to provide an additional perspective and assist in making the final decision. This rigorous, iterative process ensured an unbiased and comprehensive selection of studies for inclusion in the meta-analysis.

### 2.4 Data extraction

Two authors (BV and SS) independently conducted data extraction to ensure accuracy and reduce bias. The data collected from the full texts of the included studies encompassed several key areas:

- Studies characteristics: the first author’s name, year of publication, and the country in which the study was conducted.
- Sample sociodemographic characteristics: sample size (N), age, gender, type of diabetes (T1D or T2D), and ethnicity.
- Sample medical characteristics: duration of diabetes, levels of HbA1c, BMI, hypoglycemic episodes, the use of insulin injections, and diabetes-related hospitalizations.
- The type of control condition (e.g., waitlist/no treatment, treatment as usual (TAU), active condition).
- The type of analyses performed in the studies (intention-to-treat (ITT) or per-protocol)
- Theoretical background of the included studies.
- Measures used for evaluating the psychosocial well-being and the levels of HbA1c.
- Details of the eHealth intervention: type (e.g., smartphone-based application, web-based system), length, aim, and structure.
- Drop-out rates.
- Primary (anxiety and depression symptoms, diabetes distress and quality of life) and secondary outcomes (HbA1c).
- The mean and standard deviation of each outcome measurement at baseline, endpoint, and, if applicable, at follow-up.

Additionally, where reported, the effect size with 95% Confidence Intervals (CIs) for each outcome in both study arms was included. This comprehensive data extraction ensured that all relevant aspects of the studies were captured for analysis, enabling a thorough evaluation of the efficacy of the eHealth interventions for supporting the psychosocial well-being of adolescents with T1D and T2D.

### 2.5 Quality assessment

The Risk of Bias (RoB) for the included RCTs was independently assessed by two authors (GB and SS) using Cochrane’s Risk of Bias tool version 2 (RoB 2.0). Each study was evaluated across several critical domains: the randomization process, deviations from intended interventions (considering both the effect of assignment to the intervention and adherence to the intervention), missing outcome data, outcome measurement, and selection of reported results. Each domain was rated as “low”, “some concern”, or “high” risk based on whether the criteria were adequately met. Any discrepancies in the evaluations were resolved through thorough discussion or by consulting a third author (SS or DD). The assessments of each domain were then synthesized into an overall judgment to provide a comprehensive summary of the study’s quality.

### 2.6 Data analysis

Statistical analyses were run using R, specifically employing the *meta* (14) and *metafor* (15) packages. Outcomes at both the endpoint and follow-up were meta-analyzed when data from at least three studies were available. The effects were quantified using Standardized Mean Differences (SMD) with 95% Confidence Intervals (CI).

Heterogeneity among the studies was evaluated using the I² statistic, which measures the percentage of variation across studies that is due to heterogeneity rather than chance. I² value exceeding 50% was interpreted as indicating substantial heterogeneity, suggesting that the observed variability among study results is beyond what would be expected by chance alone. In such cases (I² > 50%), a random effects (RE) model was employed to account for the variability among study results by assuming that the effects being estimated in different studies are not identical but follow some distribution (4). The results are displayed in forest plots. Moreover, we conducted exploratory meta-analyses of quality of life subdomains where reported. For each subdomain with ≥3 studies, we applied a fixed/common-effect (FE) inverse-variance model using Hedges’ g (SMD, Hedges correction).

To assess intervention acceptability, OR with 95% CI were calculated based on drop-out rates from the studies. Drop-out rates are commonly used as an indicator of treatment acceptability as higher drop-out rates can indicate lower acceptability of the intervention by users. The ORs compared the odds of dropping out between the intervention and control groups, providing a measure of how acceptable the intervention was relative to the control.

Publication bias was assessed through two primary methods. First, visual inspection of funnel plots was conducted to identify any asymmetry, which might suggest the presence of publication bias. Asymmetry in funnel plots can indicate that smaller studies with non-significant results are less likely to be published. Second, Egger’s regression test was performed to statistically evaluate the asymmetry of the funnel plots. A significant intercept (*p* < 0.05) indicates potential publication bias. Whether evidence of publication bias was detected through these methods, further analysis was performed, relying on the trim and fill method. This method estimates the number of missing studies that might exist due to publication bias and adjusts the overall effect size accordingly (4). Additionally, the fail-safe number was calculated to determine the robustness of the meta-analysis’s results. The fail-safe number indicates how many additional studies with null results would be needed to reduce the observed effect to a non-significant level, providing an assessment of the stability and reliability of the observed effect despite potential publication bias (4).

## 3 Results

### 3.1 The search process

**Figure 1:**
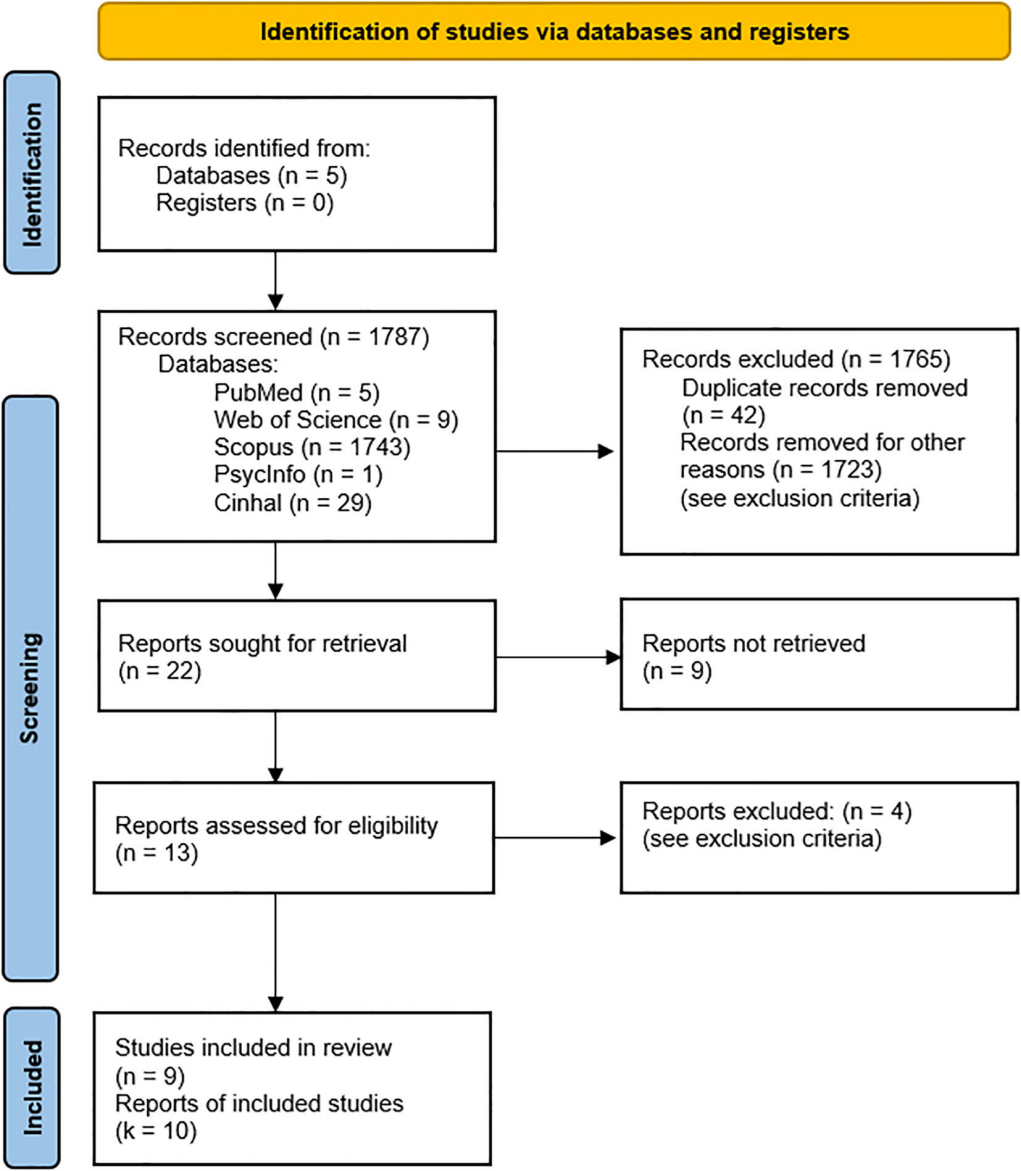
Preferred reporting items for systematic reviews and meta-analyses.

**Figure 1** displays the search process through the use of the PRISMA. The PRISMA checklist is provided within the Supplementary Material (**Supplementary Figure S2**). The initial search yielded 1,787 studies. After duplicates were removed, the titles and abstracts of 22 studies were screened resulting in 13 studies considered for full-text screening. Following the exclusion of 4 studies (see, Supplementary Material **Supplementary Table S3** for the full list of excluded studies and reasons for exclusion), 9 studies were finally included and meta-analyzed. In particular, the study of Han et al. (16) has been considered as two separate RCTs, ultimately resulting in a total of k = 10 RCTs included. In particular, a total of k = 3 studies used the Intention-to-Treat protocol (17–19), k = 1 study adopted the per-protocol (20), while k = 5 studies did not specify it (16, 21–24).

### 3.2 Descriptive statistics

**Table 1** reports the characteristics of the included studies.

**Table 1:** Characteristics of the included studies.

Authors (year)	Country	Diabetes	Sample size (experimental group)	Sample size (control group)	Age (M ± SD)	Diabetes duration in years (M ± SD)	Ethnicity (%)	Control condition (waiting list; TAU; active)	Treatment length
Ayar et al. (2021) (24)	Turkey	T1D	30	32	14.2 ± 1.76	6.01 ± 3.71	NR	TAU	~26 weeks (6 months)
Castensøe-Seidenfaden et al. (2018) (19)	Denmark	T1D	76	75	17.6 ± 2.6	8 ± 4.5	NR	TAU	~52 weeks (12 months)
Goyal et al. (2017) (23)	Canada	T1D	46	45	14 ± 1.6	6.85 ± 3.2	NR	TAU	~52 weeks (12 months)
Graue et al. (2005) (21)	Norway	T1D	45	38	14.2 ± 1.5	6.5 ± 3.6	NR	TAU	~64 weeks (15 months)
Han et al. (2015) (16)	United States	T1D	20	10	13.7 ± 1.91	5.33	Caucasians 76.7% African Americans 20% Asians 3.33%	TAU	~15 weeks (3–4 months)
Klee et al. (2018) (20)	Switzerland	T1D	20	13	13.3 ± 2.3	4.33 ± 2.9	NR	Mix of TAU - washout period and intervention	~13 weeks (3 months)
Lawson et al. (2005) (17)	Canada	T1D	23	23	15.2 ± 1.25	6.5 ± 3.25	NR	TAU	~26 weeks (6 months)
Newton et al. (2009) (18)	New Zeland	T1D	38	40	14.4 ± 2.37	NR	NR	TAU	12
Newton et al. (2013) (22)	Utah (USA)	T1D	25	25	14.5	6.25	Caucasians 96% non-Caucasians 4%	TAU	7 weeks

T1D , Type 1 Diabetes; NR , Not Reported; TAU , Treatment as Usual.

#### 3.2.1 Sample sociodemographic characteristics of the included studies

The majority of studies were conducted in the United States (k = 2; 16, 22) and Canada (k = 2; 17, 23), followed by one study each from Norway (21), Denmark (19), Turkey (24), Switzerland (20), and New Zealand (18).

A total of *N* = 624 participants were included, with *n* = 323 in the experimental group and *n* = 301 in the control group. Most control groups (k = 8) were assigned solely to treatment-as-usual (TAU), except for one study (20), which employed a randomized double-crossover design with a TAU period interspersed with a washout phase. In all studies, TAU consisted of standard diabetes care, including outpatient visits.

Participants in all included studies were adolescents with T1D, with an overall mean age of 15.09 years (SD = 1.96), ranging from 10 to 22 years. The experimental group had a mean age of 15.51 years (SD = 1.9), while the control group had a mean age of 15.43 years (SD = 1.91).

Ethnicity was reported in two studies. One study (*n* = 50) indicated that 96% of participants were Caucasian and 4% were non-Caucasian (22). Another study (n = 30) reported 76.7% Caucasian, 20% African American, and 3.33% Asian participants (16).

#### 3.2.2 Sample medical characteristics

The average duration of diabetes across participants was 6.7 years (SD = 3.56), reported by k = 8 studies (16–21; 23, 24), with the overall sample size of *n* = 546. (see **Supplementary Figures S4**, **S5** within the Supplementary Materials for percentage of the insulin usage and comparison of baseline means HbA1c and BMI levels between experimental *vs*. control groups).

At baseline, regarding severe hypoglycemic episodes, k = 3 studies specified their prevalence. Specifically, Klee et al. (20) observed severe hypoglycemic episodes in *n* = 20 participants from the experimental group and *n* = 13 from the control group; however, the authors noted no statistically significant differences between groups for mild, moderate, or severe hypoglycemic episodes. Similarly, Castensøe-Seidenfaden et al. (19) reported severe hypoglycemic episodes in *n* = 76 participants in the experimental group and *n* = 75 in the control group. Additionally, Graue et al. (21) identified severe episodes declared by *n* = 45 participants in the experimental group and *n* = 38 in the control group.

Diabetes-related hospitalizations were specified by k = 3 studies. In particular, one study observed diabetes-related hospitalizations during the 12-month study period in 22% (*n* = 76) of the experimental group and 11% (*n* = 75) of the control group (19). Another study reported no occurrences of diabetic ketoacidosis, emergency room visits, or diabetes-related hospitalizations throughout the study period (*n* = 46) (17). A further study found that, in the 6-month prior to the intervention, *n* = 2 participants in the experimental group (*n* = 45) were hospitalized for ketoacidosis, with one experiencing two episodes. During the intervention, *n* = 4 hospitalizations for ketoacidosis occurred in the experimental group, while no such events were reported in the control group (*n* = 38) either before or during the study (21).

### 3.3 Characteristics of digital solutions used for intervention

**Table 2** summarizes the characteristics of the digital solutions employed in the included studies.

**Table 2:** Detailed characteristics of digital solutions used in the included studies.

Authors (year)	Name of intervention	Type of intervention	Aim of intervention	Length months	Structure	Language
Ayar et al. (2021) (24)	Turkish Diabetes Education Program	Web-Based Platform	Enhance self-efficacy and quality of life	12 weeks	Guided, structured education	Turkish
Castensøe-Seidenfaden et al. (2018) (19)	Young with Diabetes	Smartphone-Based Application	Foster self-management and provide social interaction	12 months	Guided, Chat Function and Reminders	English, Danish
Goyal et al. (2017) (23)	Bant	Smartphone-Based Application	Increase self-monitoring of blood glucose	12 months	Unguided, Self-Directed Usage	English
Graue et al. (2005) (21)	Hybrid Approach	Hybrid	Improve self-care and health-related quality of life	15 months	Guided, Group Visits and Computer-Assisted Consultations	Norwegian
Han et al. (2015) (16)	Text Messaging System	Text Messaging	Improve symptom awareness	3 months	Unguided, Short Prompts	English
Klee et al. (2018) (20)	Webdia	Smartphone-Based Application	Enhance glycemic control and promote autonomy	3 months	Guided, Multidisciplinary Feedback	English, German, French
Lawson et al. (2005) (17)	Telephone-Based Intervention	Telephone-Based	Improve metabolic control and treatment adherence	6 months	Guided, Weekly Calls with Diabetes Nurse Educator	English
Newton et al. (2009) (18)	Motivational Text and Pedometer-Based Intervention	Text Messaging	Improve symptom recognition and increase physical activity	12 weeks	Unguided, Motivational Text and Pedometer	English
Newton et al. (2013) (22)	Diabetes Teen Talk	Web-Based Platform	Improve compliance and peer interaction	7 weeks	Guided, Structured Online Discussions	English

The interventions employed diverse delivery methods, highlighting the versatility of technology in supporting adolescents with T1D. Most of them (k = 3) implemented their interventions by means of smartphone-based applications: *Webdia* (20), *Young with Diabetes* (19), and *Bant* (23). These apps provide features, such as interactive tools, reminders, and feedback for diabetes self-management. The *Webdia* app included a multidisciplinary approach with feedback from healthcare professionals, aiming to enhance glycemic control and promote autonomy, while the *Young with Diabetes* app included a chat function, reminders, and peer support to foster self-management and provide social interaction. *Bant*, with a focus on increasing self-monitoring of blood glucose, relied on self-directed usage. The interventions lasted 3 months (20) and 12 months (19, 23). Among these, *Webdia* and *Young with Diabetes* were guided interventions, providing direct healthcare involvement, while *Bant* was unguided, relying on self-directed usage. Moreover, k = 2 studies adopted text messaging systems designed to improve symptom awareness (16) and a motivational text and pedometer-based intervention (18). These interventions emphasized the accessibility and convenience of short and structured prompts, aiming to improve symptom recognition and increase physical activity. Both studies (16, 18) were unguided interventions that did not involve real-time interaction with healthcare providers, and they lasted 3 months (16) and 12 weeks (18). Additionally, k = 2 studies employed web-based platforms, namely *Diabetes Teen Talk* (22) and a Turkish diabetes education program (24). These platforms delivered educational content and engagement through online discussions and problem-solving activities. *Diabetes Teen Talk* aimed to improve compliance and peer interaction, while the Turkish diabetes program sought to enhance self-efficacy and quality of life through structured education. Both the above-mentioned studies were guided interventions with professional facilitation, particularly through structured online discussions or educational guidance, lasting 7 weeks for the intervention (22) and a 12-week program (24). Lastly, one study relied on telephone-based interventions for ongoing support and guidance. In particular, Lawson et al. (17) implemented weekly calls by a diabetes nurse educator as part of a 6-month guided intervention, focusing on insulin dose adjustments and education to improve metabolic control and treatment adherence. Lastly, a hybrid approach was used by Graue et al. (21), combining group visits with computer-assisted consultations. This 15-month guided program focused on peer and family support alongside structured discussions with healthcare professionals, aiming to improve self-care and health-related quality of life. The primary language of these interventions was predominantly English (20; 16–18, 22, 23), but other languages, including Norwegian (21), Turkish (24), Danish (19), German and French (20) were included.

### 3.4 Theoretical background and approaches adopted by the included studies

The theoretical frameworks underpinning the included studies were specified by k = 6 studies, reflecting diverse approaches to addressing T1D management. One study (22) employed Bandura’s theory of self-efficacy as its foundation. Self-efficacy refers to an individual’s belief in their ability to perform specific tasks and behaviors necessary to achieve desired outcomes (25). In the context of T1D management, this theory emphasizes empowering individuals to build confidence in their capacity to effectively manage their condition. The above-mentioned study focused on interventions designed to enhance participants’ self-efficacy, fostering greater competence in handling daily diabetes-related challenges, such as blood glucose monitoring, insulin administration, and dietary adjustments. Another study (16) was based on the Health Belief Model (HBM; 26), a theoretical framework that explores the cognitive processes influencing health-related behaviors. The HBM seeks to explain under what conditions individuals are motivated to engage in preventive health measures or seek treatment. This model considers factors, such as perceived susceptibility, perceived severity of health condition, perceived benefits of taking action, and barriers to action. Han et al. (16) adopted HBM to design an intervention involving text messages.

Other studies (k = 4) also employed distinct frameworks and conceptual approaches to guide their interventions. Castensøe-Seidenfaden et al. (19) adopted a participatory design approach in developing the *Young with Diabetes* mobile health (mHealth) app, which emphasized peer support and self-management strategies tailored to the psychosocial needs of adolescents. Similarly, Goyal et al. (23) applied behavior change techniques through gamification within their *Bant* app to encourage consistent self-monitoring and diabetes management behaviors. Graue et al. (21) implemented a structured educational and psychosocial intervention that combined peer support and family engagement to enhance coping skills and self-management among adolescents. Furthermore, Klee et al. (20) evaluated the impact of a patient-designed app, *Webdia*, which incorporated self-management principles and tailored educational feedback to improve glycemic control. These diverse theoretical foundations highlight the multifaceted nature of T1D interventions, integrating behavioral, technological, and psychosocial components to address the complex needs of individuals managing this condition.

### 3.5 Self-report questionnaires used for evaluating psychosocial well-being

The reviewed studies (k = 8) adopted various psychosocial measures to evaluate quality of life and (k = 2) diabetes distress among adolescents with T1D. These measures employed a range of validated self-report questionnaires, offering robust tools to assess health-related outcomes within this population.

The most frequently used instrument for assessing quality of life was the Diabetes Quality of Life for Youth Questionnaire (DQOLY), employed in k = 5 studies (16, 17, 20, 22, 23);. This 52-item questionnaire evaluates health-related quality of life across three domains: diabetes life satisfaction, disease impact, and disease-related worries, alongside a single-item health perception measure. Responses are rated on a Likert scale, with lower scores reflecting poorer quality of life outcomes. Additionally, the Diabetes Quality of Life Questionnaire (DQOL) was used in one study (21). Originally designed for adults, this measure was adapted to assess adolescents’ satisfaction, impact, diabetes worry, and social/vocational concerns, with excellent reliability (α = 0.78–0.92). Another study (18) used the Comprehensive Quality of Life Scale–School Version, a general quality of life tool evaluating multiple life domains for adolescents. The Pediatric Quality of Life Inventory (PedsQL) 3.0 Diabetes Module (27) was employed in one study (24) to assess diabetes-specific quality of life. This 28-item multidimensional scale focuses on symptoms, treatment barriers, adherence, worry, and communication, using a 4-point Likert scale, with excellent internal consistency (α = 0.88).

As regards diabetes distress, k = 2 studies administered the Problem Areas in Diabetes (PAID) Updated Version (16, 19). This 20-item tool evaluates emotional states related to T1D, demonstrating high internal consistency (α = 0.95).

### 3.6 Measures used for assessing HbA1c levels

At baseline, HbA1c levels were assessed across the studies using standardized methods to evaluate participants’ glycemic control and eligibility for interventions. In Ayar et al. (24), HbA1c values were measured during routine clinical assessments, though specific laboratory techniques were not detailed. Castensøe-Seidenfaden et al. (19) determined HbA1c levels during clinical visits, requiring a baseline of ≥64 mmol/mol (8.0%) at the last visit or an average >58 mmol/mol (7.5%) over the three preceding visits, reflecting consistent suboptimal control. Similarly, Goyal et al. (23) included participants with HbA1c levels between 8.0% and 10.5%, measured *via* routine laboratory tests at participating endocrinology centers. Graue et al. (21) recorded baseline HbA1c with a mean of 9.3% (range: 6.1–12.8%) using standardized clinical methods. Han et al. (16) also used routine laboratory assessments to measure HbA1c, focusing on adolescents with levels >8.5% over the previous 6 months. In Klee et al. (20), HbA1c was measured using laboratory-certified methods, including participants with values above 8.0%, adhering to clinical standards for accuracy. Lawson et al. (17) similarly employed standardized laboratory assays, including participants with HbA1c >8.5% over 6 months, ensuring a consistent baseline of poor glycemic control. Finally, Newton et al. (18) used clinical evaluations to measure baseline HbA1c before interventions, though specific techniques were not detailed. Across all studies, the use of standardized laboratory methods, such as HPLC or immunoassays, ensured accurate and reliable baseline assessments critical for evaluating intervention outcomes.

### 3.7 Risk of bias

**Figure 2:**
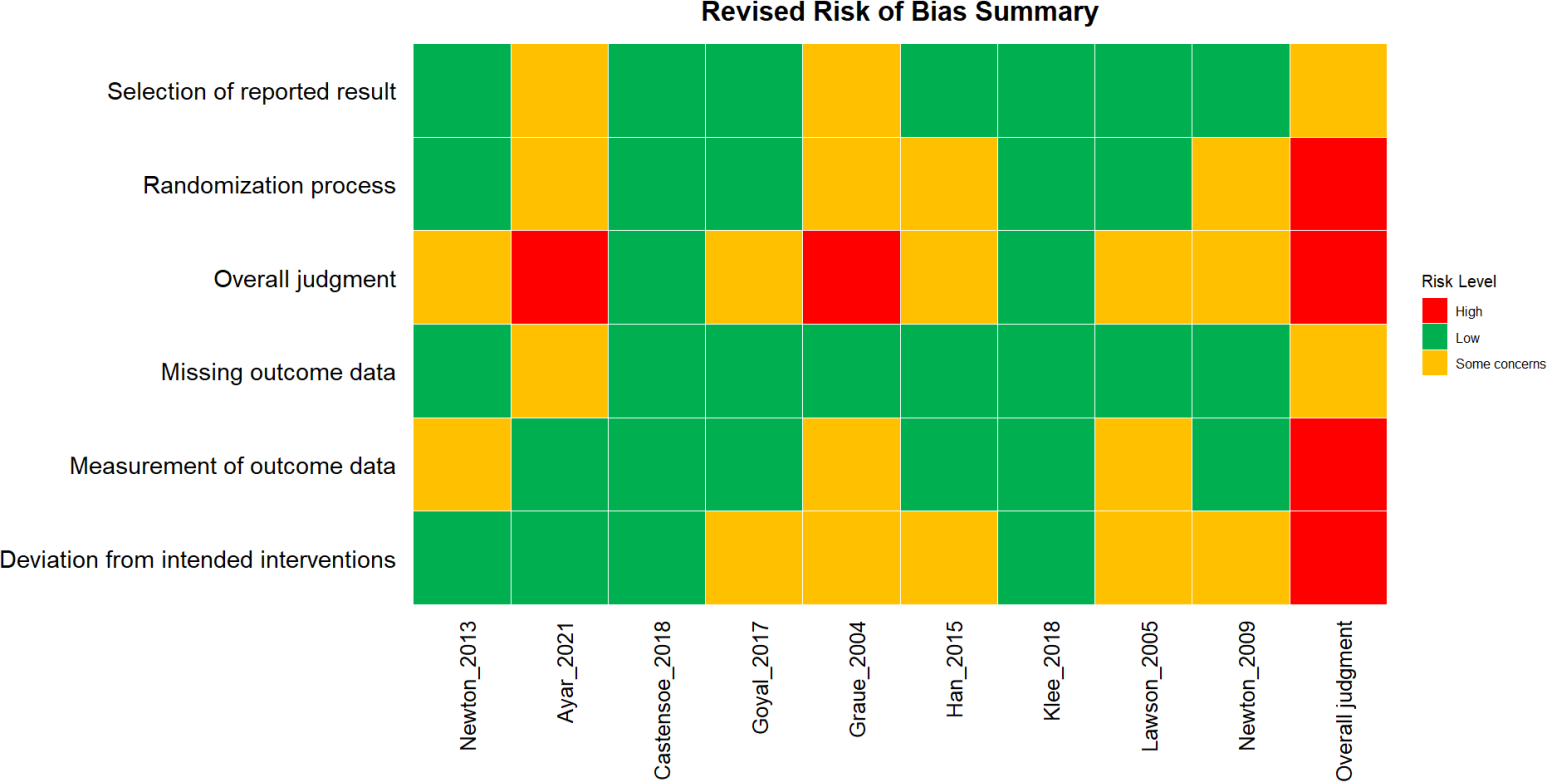
Risk of Bias of the included studies.

As shown in **Figure 2**, overall, the quality of the studies revealed that k = 6 studies raised “some concerns” while k = 2 studies “high concerns” regarding RoB. Only one study demonstrated a low risk of bias (19). The main sources of bias were related to deviations from the intended interventions, selection of reported results, and issues with the randomization process. Specifically, the domain investigating deviations from the intended interventions showed concern in 42.85% of the studies, with an additional 14.28% classified as high risk. This was primarily due to inadequate controls over adherence to protocols in the experimental groups. The domain examining the selection of reported results highlighted concerns in 71.4% of the studies, where the absence of a predefined protocol or clear specification of planned analyses led to significant uncertainty, though none of the studies reached a high risk in this area.

Regarding the randomization process, half of the studies were rated as low risk, while 14.28% demonstrated a high risk due to insufficient reporting on sequence generation or allocation concealment. Lastly, in the domain of measurement of outcome data, 42.85% of the studies showed some concerns, often arising from a lack of blinding or inconsistencies in measurement across study arms. Overall, deviations from intended interventions and the selection of reported results emerged as the most frequent source of bias.

### 3.8 Results on the efficacy of eHealth interventions

None of the included studies evaluated symptoms of depression or anxiety, therefore no analyses have been performed. Only two studies (16, 19) assessed diabetes-related distress, using the Problem Areas in Diabetes (PAID) scale. While this tool effectively measures the emotional burden associated with diabetes management, the limited number of studies employing it precluded any meaningful meta-analyses or comparative evaluations of distress outcomes. Moreover, follow-up could not be evaluated due to lack of data.

#### 3.8.1 Quality of Life in adolescents with T1D

**Figure 3:**
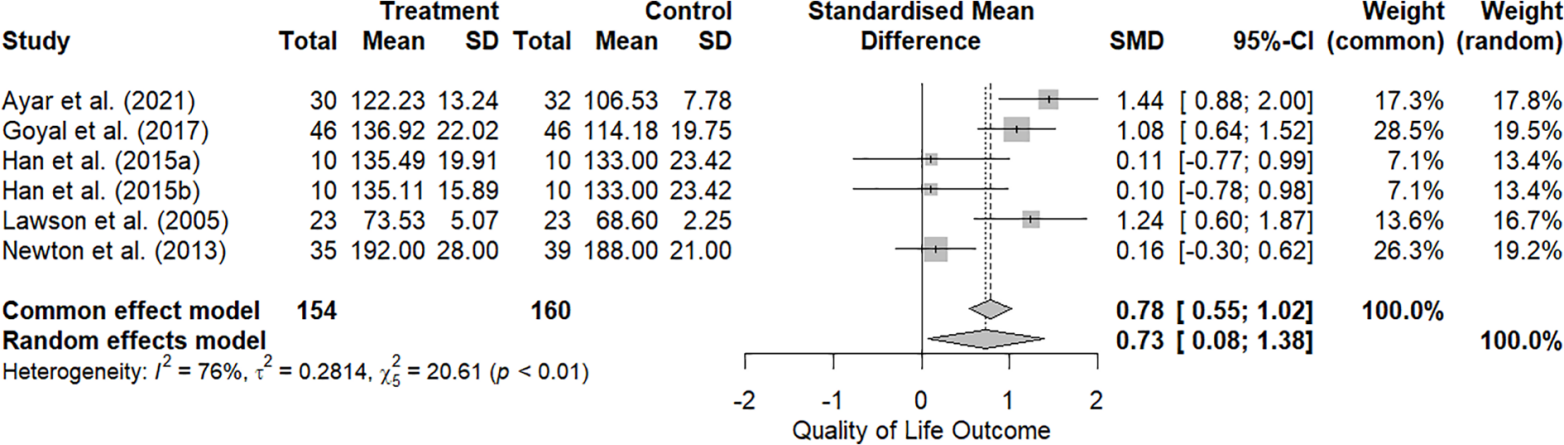
Forest plot of effect sizes for quality of life in adolescents with T1D (k = 6 studies).

As shown in **Figure 3**, the RE model results for quality of life indicated substantial heterogeneity among studies, with a tau² of 0.28 and an I² of 75.7%. The Q-test for heterogeneity was significant (*p* < 0.01), supporting the presence of variability across study findings. The pooled effect size was 0.73, statistically significant (95% CI [0.08, 1.38]; *p* < 0.01), suggesting a moderate positive effect of the interventions on QoL in adolescents with T1D.

#### 3.8.2 Quality of Life in adolescents with T1D: satisfaction with life, impact of diabetes and worries about diabetes subscales of DQOLY

**Figure 4:**
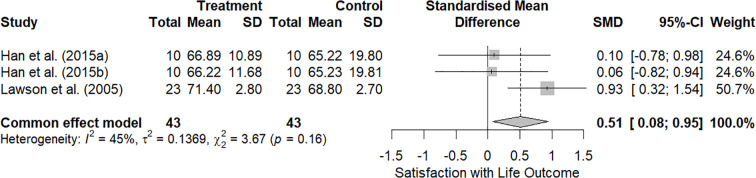
Forest plot of effect sizes for Satisfaction with life in adolescents with T1D (k = 3 studies).

A FE meta-analysis of three studies (k = 3) indicated a moderate, statistically significant improvement in satisfaction with life in using eHealth solutions versus control groups (SMD = 0.51, 95% CI [0.08, 0.95]; z = 2.30, p = .022), as shown in **Figure 4**. Between-study heterogeneity was moderate and not significant (I² = 45%; Q(2) = 3.67, p = .16).

By contrast, the impact of diabetes and worries about diabetes subdomains were not significant; detailed results are provided in the Supplementary Materials (**Supplementary Figures S6**, **S7**).

#### 3.8.3 Hemoglobin A1c

**Figure 5:**
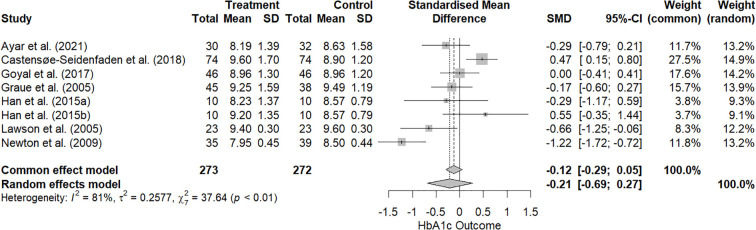
Forest plot of effect sizes for HbA1c in adolescents with T1D (k = 8 studies).

As shown in **Figure 5**, the RE model for HbA1c revealed a tau² estimate of 0.26 and an I² of 81.4%, indicating substantial heterogeneity across studies. The Q-test for heterogeneity was significant (*p* < 0.01), confirming the presence of considerable variability. The overall effect size was -0.21, which shows a small decrease, however, not statistically significant (95% CI = -0.69–0.27; *p* = 0.34).

#### 3.8.4 Publication bias through Egger’s test

**Figure 6:**
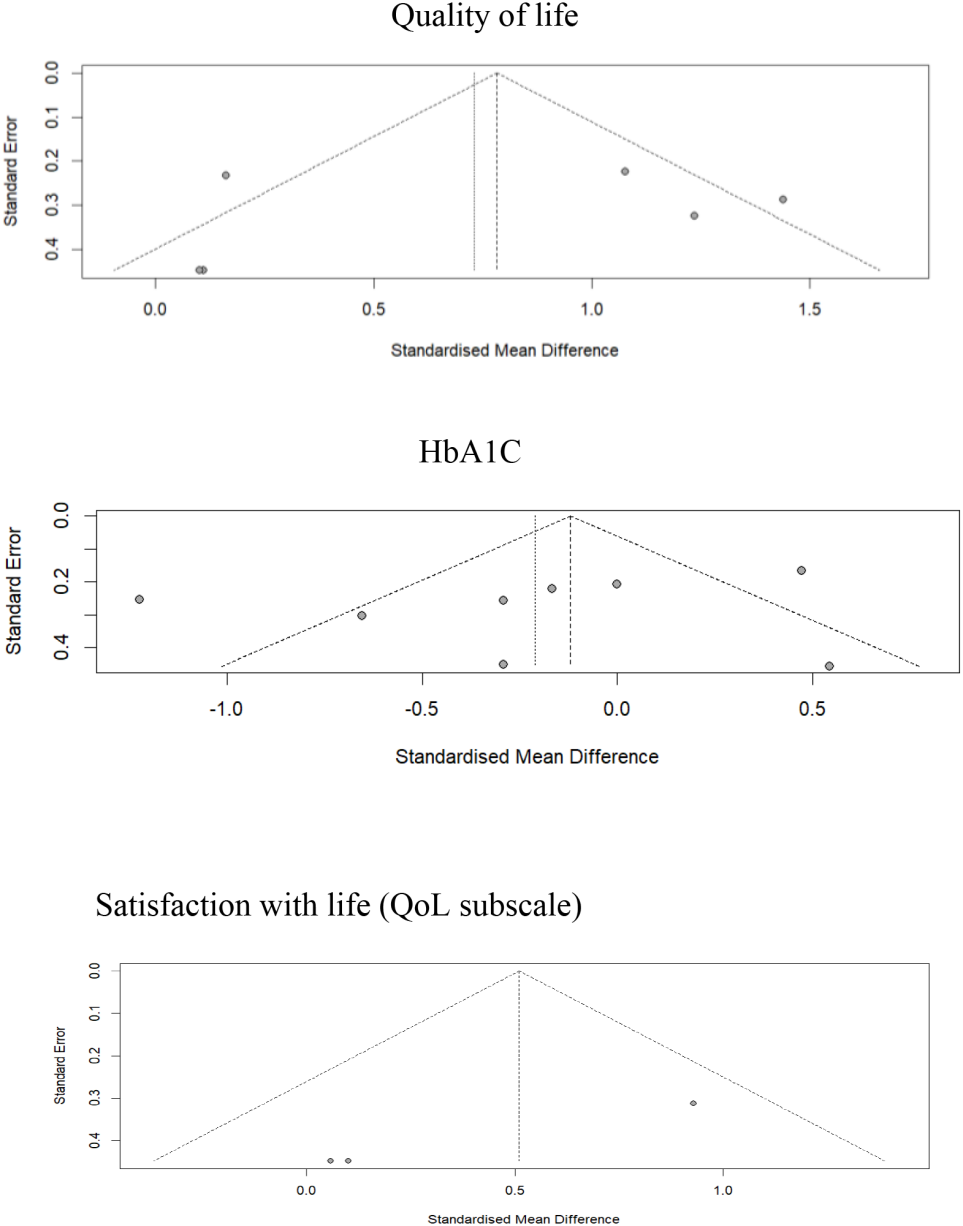
Funnel plots assessing publication bias for quality of life and HbA1c outcomes.

Publication bias was assessed for all outcomes, as shown in **Figure 6**. Egger’s regression test revealed no significant publication bias for HbA1c (t = -0.89, *p* = 0.41) or quality of life (t = -0.49, *p* = 0.65), suggesting a low risk of publication bias impacting these results. Consequently, additional bias-adjustment procedures, such as trim-and-fill, were deemed unnecessary, as the funnel plots showed no significant asymmetry. On contrary, visual inspection of the funnel plot of satisfaction with life subscale suggested some asymmetry. Egger’s regression was statistically significant (t = −28.02, df = 1, p = .023); however, given the very small number of studies (k = 3), tests of small-study effects are underpowered and unstable.

In **Supplementary Figure S8** and **Supplementary Figure S9** within the Supplementary Materials are shown the funnel plots of the DQOLY subscales, i.e., impact of diabetes and worries about diabetes.

##### 3.9 Odds ratio

The meta-analysis results indicate no significant heterogeneity among the studies (I² = 0.00%), suggesting that variability in effect sizes is likely due to sampling variability rather than real differences between studies. The estimated effect size (log odds ratio) was 0.47 (95%CI, -0.07–1.01, *p* = 0.09), indicating that the effect was not statistically significant. Overall, these findings suggest no significant difference in dropout rates between the experimental and control groups across the included studies. This conclusion is supported by the forest and funnel plots, with the latter also showing no evidence of publication bias.

## 4 Discussion

### 4.1 The impact of eHealth interventions on psychosocial and medical outcomes in adolescents with T1D

This meta-analysis assessed the impact of eHealth interventions on psychosocial and medical outcomes among adolescents with T1D. Specifically, the primary objective was to evaluate the effectiveness of these digital interventions in alleviating anxiety and depression symptoms, and diabetes distress as well as in improving the overall quality of life. The secondary objective was to assess their impact on glycemic control (HbA1c).

In particular, this meta-analysis incorporated data from 10 RCTs involving 624 participants with T1D, aged 10–22 years, with an overall mean age of 15.09 (SD = 1.96). The inclusion of this extended age range was motivated by the characteristics of two studies that included participants from 10 years old (20) and up to 22 years old (16), while the mean age across studies remained consistent with the adolescent demographic (mean age equal to 13.3 and 13.7, respectively). Adolescence and early adulthood represent critical developmental stages marked by physical, psychological, and social changes that are further exacerbated by the challenges of managing a chronic condition like T1D (43). Including this range allowed for a comprehensive evaluation of eHealth interventions across this transitional period. Importantly, the inclusion of older adolescents aligns with the shared psychosocial and medical needs of these populations, which often overlap due to the gradual transition to independence in diabetes self-management (28).

Although exploratory, several participant/clinical features could plausibly shape psychosocial or glycemic responses to eHealth (e.g., insulin delivery modality, baseline HbA1c and BMI levels). For instance, pump users may engage differently with digital feedback than those on multiple daily injections (29); higher baseline HbA1c may be associated with larger room for improvement (30); and BMI could correlate with activity-focused components (31). However, across the included studies these variables were inconsistently operationalized, and the small number of studies precluded reliable moderator/meta-regression analyses.

None of the included studies specifically targeted adolescents with T2D. This may suggest that research on eHealth interventions has largely focused on T1D in adolescents, possibly due to its higher prevalence and earlier onset compared to T2D, which is often associated with lifestyle factors and historically considered rare in younger populations (32, 33). Moreover, adolescents with T1D frequently experience psychological challenges, including diabetes distress, depression and anxiety symptoms, which can significantly impact their overall well-being and diabetes management (13, 34). Although this meta-analysis also focused on anxiety and depressive symptoms, it is noteworthy that only a limited number of RCTs have investigated these outcomes in adolescents with T1D. However, these studies addressing depressive symptoms could not be included due to incomplete reporting of data (e.g., absence of means and standard deviations). This limitation underscores a significant gap in literature and highlights the need for future research to ensure comprehensive reporting, facilitating more robust quantitative analyses in this critical area. Research indicates that approximately 22.2% of children with T1D experience depression, and 17.7% experience anxiety (35), emphasizing the clinical salience of these outcomes and the importance of their systematic measurement. For instance, in the included studies, Han et al. (16) and Castensøe-Seidenfaden et al. (19) evaluated diabetes distress, which is a condition distinct from depression and anxiety, and affects about one-third of adolescents with T1D and is associated with suboptimal glycemic control and reduced self-care behaviors (36). This conceptual distinction is important: focusing exclusively on the distress related to diabetes can lead to overlooking broader internalized symptoms that carry an independent risk of poorer adherence to treatment and poorer quality of life. Therefore, it should be crucial to assess depression and anxiety symptoms, and diabetes distress as distinct constructs. Notwithstanding this, the findings of this meta-analysis demonstrated varied effects of these digital tools on quality of life, satisfaction with life, and HbA1c outcomes, three critical outcomes in the management of T1D, further highlighting the complexity and variability of eHealth interventions’ impact on adolescents with T1D.

In terms of quality of life, and of satisfaction with life as a subscale of the overall construct, the eHealth interventions demonstrated a significant and moderately positive impact, highlighting the potential of digital tools to meaningfully enhance the psychosocial well-being of adolescents managing T1D. This positive effect emphasizes the important role of digital interventions in supporting overall quality of life, a crucial factor for adolescents coping with the complexities of a chronic condition (37). Although substantial heterogeneity was observed across studies, it was likely stemming from differences in the design and delivery of interventions, sample characteristics, and measurement tools (38). For instance, tools, such as the DQOLY and the PedsQL questionnaires were used to assess quality of life, but differences in their focus on diabetes-specific versus general domains may contribute to discrepancies in reported outcomes. Moreover, differences in the design and theoretical grounding of digital solutions likely contributed to the observed pattern of psychosocial outcomes. Interventions explicitly rooted in self-efficacy theory (e.g., 22) or the Health Belief Model (e.g., 16) targeted core mechanisms, such as confidence in diabetes self-management and perceived barriers/beliefs, which may be linked to improvements in coping skills and psychosocial well-being. Programs that are guided and combined peer support and professional feedback, such as Young with Diabetes (19), Webdia (20), and Diabetes Teen Talk (22), further reinforced emotional regulation and social interaction, contributing to improvements in quality of life.

The analysis regarding HbA1c revealed no statistically significant overall effect despite a small trend toward improvement. The use of HbA1c as a primary medical outcome is critical, as it reflects long-term glycemic control. However, the absence of significant findings underscores the complexity of diabetes management, where psychosocial and behavioral factors interplay with medical outcomes. Notably, our results on HbA1c contrast with prior meta-analyses that reported significant reductions in HbA1c. Liu et al. (28) found an overall decrease in HbA1c across 26 studies, particularly when interventions included professional feedback. Similarly, Bonoto et al. (39) observed significant improvements in patients with T1D and T2D using mHealth apps that combined multiple self-management features with professional support. By contrast, our review was limited to adolescents with T1D, a population facing distinctive developmental and adherence challenges, and included several unguided and relatively short interventions. These differences in population, intervention design, and level of professional involvement may explain the absence of significant effects on HbA1c despite the psychosocial benefits observed. Moreover, the limited effect may be attributed to the high heterogeneity in intervention duration and characteristics, participants’ adherence rates, and baseline glycemic control across studies, which could have diluted potential effects (e.g., 21-24). This further suggests that eHealth interventions may require longer implementation periods or more structured approaches to meaningfully influence medical outcomes, underscoring opportunities for refinement in future studies.

### 4.2 The role of eHealth solutions: their acceptability and engagement

The interventions employed a variety of digital solutions, reflecting the versatility of technology in supporting adolescents with T1D. Smartphone-based applications, such as *Webdia* (20), *Young with Diabetes* (19), and *Bant* (23), were among the most frequently used. These apps provide interactive tools, reminders, and feedback to facilitate self-management. Guided interventions, such as *Webdia* and *Young with Diabetes*, incorporated peer support and professional healthcare feedback, whereas unguided interventions, like *Bant*, relied on self-directed use.

Other technologies included text messaging systems, as in the motivational intervention and the symptom-awareness system, both implemented by Newton et al. (22) and by Han et al. (16), respectively. Web-based platforms, such as *Diabetes Teen Talk* (22), emphasized peer interaction and problem-solving, while hybrid approaches like integrated group visits and computer-assisted consultations (21). These diverse approaches highlight the potential of eHealth to address both psychosocial and medical needs through tailored delivery methods.

The analysis of drop-out rates between intervention and control groups revealed no significant differences, suggesting good overall acceptability of eHealth interventions among adolescent participants. Nonetheless, engagement can be negatively influenced by tool familiarity and academic demands such as exams and extracurricular activities (22), device-related burdens, such as carrying a second phone or using incompatible adapters (23), technical issues, such as app responsiveness (20), and greater health burdens (e.g., low self-esteem; 21). Lastly, short-lived interest in tools, such as pedometers and limited engagement strategies contributed to declining participation (18). These findings align with existing literature, showing similar dropout rates across study arms. For instance, a systematic review and meta-analysis examining smartphone-based diabetes rehabilitation apps among adults and adolescents found comparable dropout rates between experimental and control groups, indicating that such digital interventions are generally well-received by participants (40). High acceptability is crucial for supporting ongoing engagement and long-term effectiveness, especially for adolescents who also have to manage a chronic condition, such as T1D.

### 4.3 Limitations and future developments

The results of this meta-analysis should be interpreted in light of several limitations. The limited availability of studies that integrate psychosocial factors into digital health solutions precluded more detailed or stratified analyses on specific parameters (41, 42). Moreover, the present meta-analysis could not analyze depression and anxiety symptoms, as well as diabetes distress due to limited data. Given the distinct and complex mechanisms underlying in particular depression and anxiety, it is possible that one symptom may show improvement while the other does not. While often co-occurring, they may respond differently to interventions due to differences in type, designs, duration, and the specific mental health symptoms targeted. This variability underscores the importance of future research that includes larger sample sizes and more comprehensive reporting on these constructs separately. Furthermore, the majority of included studies were conducted in high-income countries, which may limit the generalizability of findings to adolescents in low- and middle-income settings. Differences in access to technology, healthcare infrastructure, and socioeconomic factors may influence the effectiveness of eHealth interventions. Future research should explore the applicability of these interventions in different economic and cultural contexts, ensuring equitable access and effectiveness across populations. Additionally, few studies provided follow-up assessments, making it difficult to evaluate the sustainability of improvements over time. This limitation highlights an important gap in the existing literature, as the effectiveness of eHealth interventions may diminish once participants discontinue their use or face changes in their environment, such as transitioning to adulthood or experiencing different life circumstances. To better understand long-term effects, future trials should incorporate extended follow-up periods, such as six months or one year, to assess whether improvements are sustained over time. Understanding the duration of effectiveness will provide valuable insights into the optimal timing and duration of digital interventions, ensuring that they can be designed in order to offer lasting support in managing T1D. The present meta-analysis explored a very new topic and further research in this field is needed to allow for a more nuanced understanding of these interventions. Given the growing number of adolescents affected by T2D, it is important that upcoming studies also focus on this population to evaluate how digital health tools may support their unique clinical and psychosocial needs. Future research should also focus on adopting RCT designs to validate the efficacy of eHealth interventions while considering the interplay between psychosocial and medical factors. In particular, fear of hypoglycemia (FoH), although not pre-specified in our objectives, should be explicitly included as a key domain when evaluating diabetes-related psychosocial functioning. FoH is common in youth with T1D and is linked to avoidance behaviors (e.g., maintaining higher glucose targets or under-dosing insulin), poorer quality of life, and suboptimal glycemic outcomes (43, 44). In particular, exploring interventions of longer duration and those that incorporate multiple behavior-change components may help achieve more meaningful changes in clinical outcomes. Moreover, a deeper understanding of the factors that influence patients’ acceptance and engagement with eHealth technologies could be crucial for designing more effective interventions. Emerging approaches, such as personalization through artificial intelligence or the use of gamification features to encourage user participation may further enhance engagement and treatment success among adolescents. This includes examining the role of user interface design, ease of use, and perceived usefulness in enhancing adolescents’ engagement and outcomes. For instance, ensuring that eHealth tools are compatible with a wide range of existing devices and platforms can improve usability and adoption by reducing barriers related to device requirements or setup. Integrating subjective reports with objective, real-time physiological data—such as continuous glucose monitoring or stress biomarkers—may provide a more holistic understanding of intervention impact. Subgroup analyses of eHealth intervention effectiveness could also be valuable, as they may reveal differential impacts across diverse demographic or clinical populations, allowing for more personalized and targeted approaches. In addition, employing validated tools to assess psychological distress would offer a more complete picture of participants’ mental health and better inform intervention design.

## 5 Conclusion

This meta-analysis sheds light on the expanding evidence base surrounding eHealth interventions for adolescents with T1D, offering critical insights into their potential benefits. The findings underscore the significant role these digital tools, particularly smartphone-based apps, can play in improving quality of life and satisfaction with life, an additional component to the traditional care in supporting the T1D management in managing T1D effectively. Adolescence represents a uniquely challenging phase, marked by the interplay of psychosocial development and the demanding self-management of diabetes. Enhancing quality of life and satisfaction with life is therefore a crucial outcome, as it reflects improved psychosocial well-being and may promote better engagement and adherence to diabetes over time. Future studies could focus on integrating psychosocial aspects, such as diabetes distress, depression and anxiety symptoms along with robust medical management to offer comprehensive care. Such approaches have the potential to enhance both physical health and affective well-being, providing adolescents with integrated support - in presence and online - in navigating the complexities of T1D.

## Data Availability

The original contributions presented in the study are included in the article/**Supplementary Material**. Further inquiries can be directed to the corresponding author.
