# Supplementary material for: Evaluating the impact of eHealth interventions on adolescents with diabetes: a systematic review and meta-analysis
Source: Front Clin Diabetes Healthc. 2025 Dec 10;6:1659146. doi: 10.3389/fcdhc.2025.1659146 (PMC12728833; doi:10.3389/fcdhc.2025.1659146)
Supplement: Supplementary file 1 [file DataSheet1.docx]

Supplementary Material

***Table S1.*** *Search string for each database*

Exact keyword search strategies used across databases for studies.

| **Database** | **Search String** |
| --- | --- |
| **PubMed** | ("type 1 diabetes" OR "type 2 diabetes" OR T1D OR T2D) AND (ehealth OR mhealth OR "mobile health" OR smartphone* OR app OR apps OR application* OR telehealth OR telemedicine OR telemonitor* OR "text messag*" OR sms OR "short message*" OR web-based OR internet OR online OR "digital game*" OR gamif*) AND (adolescen* OR teen* OR youth OR "young adult*") AND ("quality of life" OR qol OR depression OR depressive OR anxiety OR anxious OR "diabetes distress" OR distress OR paid OR pedsql OR dqol OR dqoly OR hba1c OR glyc* OR glucose) AND (random* OR trial OR "randomized controlled trial") |
| **Scopus** | TITLE-ABS-KEY(("type 1 diabetes" OR "type 2 diabetes" OR T1D OR T2D) AND (ehealth OR mhealth OR "mobile health" OR smartphone* OR app OR apps OR application* OR telehealth OR telemedicine OR telemonitor* OR "text messag*" OR sms OR "short message*" OR web-based OR internet OR online OR "digital game*" OR gamif*) AND (adolescen* OR teen* OR youth OR "young adult*") AND ("quality of life" OR qol OR depression OR depressive OR anxiety OR anxious OR "diabetes distress" OR distress OR paid OR pedsql OR dqol OR dqoly OR hba1c OR glyc* OR glucose) AND (random* OR trial OR "randomized controlled trial")) |
| **Web Of Science** | TS=(("type 1 diabetes" OR "type 2 diabetes" OR T1D OR T2D) AND (ehealth OR mhealth OR "mobile health" OR smartphone* OR app OR apps OR application* OR telehealth OR telemedicine OR telemonitor* OR "text messag*" OR sms OR "short message*" OR web-based OR internet OR online OR "digital game*" OR gamif*) AND (adolescen* OR teen* OR youth OR "young adult*") AND ("quality of life" OR qol OR depression OR depressive OR anxiety OR anxious OR "diabetes distress" OR distress OR paid OR pedsql OR dqol OR dqoly OR hba1c OR glyc* OR glucose) AND (random* OR trial OR "randomized controlled trial")) |
| **PsycINFO** | TI,AB,KW(("type 1 diabetes" OR "type 2 diabetes" OR T1D OR T2D) AND (ehealth OR mhealth OR "mobile health" OR smartphone* OR app OR apps OR application* OR telehealth OR telemedicine OR telemonitor* OR "text messag*" OR sms OR "short message*" OR web-based OR internet OR online OR "digital game*" OR gamif*) AND (adolescen* OR teen* OR youth OR "young adult*") AND ("quality of life" OR qol OR depression OR depressive OR anxiety OR anxious OR "diabetes distress" OR distress OR paid OR pedsql OR dqol OR dqoly OR hba1c OR glyc* OR glucose) AND (random* OR trial OR "randomized controlled trial")) |
| **CINAHL** | TI ("type 1 diabetes" OR "type 2 diabetes" OR T1D OR T2D ) AND AB ( ehealth OR mhealth OR "mobile health" OR smartphone* OR app OR apps OR application* OR telehealth OR telemedicine OR telemonitor* OR "text messag*" OR sms OR "short message*" OR web-based OR internet OR online OR "digital game*" OR gamif* )AND TI,AB ( adolescen* OR teen* OR youth OR "young adult*" ) AND TI,AB ( "quality of life" OR qol OR depression OR depressive OR anxiety OR anxious OR" diabetes distress" OR distress OR paid OR pedsql OR dqol OR dqoly OR hba1c OR glyc* OR glucose )AND TI,AB ( random* OR trial OR "randomized controlled trial") |

*Figure S2. PRISMA checklist*

| **Section and Topic** | **Item #** | **Checklist item** | **Location where item is reported** |
| --- | --- | --- | --- |
| **TITLE** | | |  |
| Title | 1 | Identify the report as a systematic review. | 1 |
| **ABSTRACT** | | |  |
| Abstract | 2 | See the PRISMA 2020 for Abstracts checklist. | 1 |
| **INTRODUCTION** | | |  |
| Rationale | 3 | Describe the rationale for the review in the context of existing knowledge. | 2 |
| Objectives | 4 | Provide an explicit statement of the objective(s) or question(s) the review addresses. | 2-3 |
| **METHODS** | | |  |
| Eligibility criteria | 5 | Specify the inclusion and exclusion criteria for the review and how studies were grouped for the syntheses. | 3 |
| Information sources | 6 | Specify all databases, registers, websites, organisations, reference lists and other sources searched or consulted to identify studies. Specify the date when each source was last searched or consulted. | 3 |
| Search strategy | 7 | Present the full search strategies for all databases, registers and websites, including any filters and limits used. | 3 |
| Selection process | 8 | Specify the methods used to decide whether a study met the inclusion criteria of the review, including how many reviewers screened each record and each report retrieved, whether they worked independently, and if applicable, details of automation tools used in the process. | 3-4 |
| Data collection process | 9 | Specify the methods used to collect data from reports, including how many reviewers collected data from each report, whether they worked independently, any processes for obtaining or confirming data from study investigators, and if applicable, details of automation tools used in the process. | 3-4 |
| Data items | 10a | List and define all outcomes for which data were sought. Specify whether all results that were compatible with each outcome domain in each study were sought (e.g. for all measures, time points, analyses), and if not, the methods used to decide which results to collect. | 5 |
|  | 10b | List and define all other variables for which data were sought (e.g. participant and intervention characteristics, funding sources). Describe any assumptions made about any missing or unclear information. | 5 |
| Study risk of bias assessment | 11 | Specify the methods used to assess risk of bias in the included studies, including details of the tool(s) used, how many reviewers assessed each study and whether they worked independently, and if applicable, details of automation tools used in the process. | 5 |
| Effect measures | 12 | Specify for each outcome the effect measure(s) (e.g. risk ratio, mean difference) used in the synthesis or presentation of results. | 5 |
| Synthesis methods | 13a | Describe the processes used to decide which studies were eligible for each synthesis (e.g. tabulating the study intervention characteristics and comparing against the planned groups for each synthesis (item #5)). | 5 |
|  | 13b | Describe any methods required to prepare the data for presentation or synthesis, such as handling of missing summary statistics, or data conversions. | 5 |
|  | 13c | Describe any methods used to tabulate or visually display results of individual studies and syntheses. | 5 |
|  | 13d | Describe any methods used to synthesize results and provide a rationale for the choice(s). If meta-analysis was performed, describe the model(s), method(s) to identify the presence and extent of statistical heterogeneity, and software package(s) used. | 5 |
|  | 13e | Describe any methods used to explore possible causes of heterogeneity among study results (e.g. subgroup analysis, meta-regression). | 5 |
|  | 13f | Describe any sensitivity analyses conducted to assess robustness of the synthesized results. | 5 |
| Reporting bias assessment | 14 | Describe any methods used to assess risk of bias due to missing results in a synthesis (arising from reporting biases). | 5 |
| Certainty assessment | 15 | Describe any methods used to assess certainty (or confidence) in the body of evidence for an outcome. | NA |
| **RESULTS** | | |  |
| Study selection | 16a | Describe the results of the search and selection process, from the number of records identified in the search to the number of studies included in the review, ideally using a flow diagram. | 6 & Figure 1 |
|  | 16b | Cite studies that might appear to meet the inclusion criteria, but which were excluded, and explain why they were excluded. | 6 & Figure S1 (supplementary materials) |
| Study characteristics | 17 | Cite each included study and present its characteristics. | 7-13 |
| Risk of bias in studies | 18 | Present assessments of risk of bias for each included study. | 14 |
| Results of individual studies | 19 | For all outcomes, present, for each study: (a) summary statistics for each group (where appropriate) and (b) an effect estimate and its precision (e.g. confidence/credible interval), ideally using structured tables or plots. | 15-16 |
| Results of syntheses | 20a | For each synthesis, briefly summarise the characteristics and risk of bias among contributing studies. | 7-11 (Table 1),14 |
|  | 20b | Present results of all statistical syntheses conducted. If meta-analysis was done, present for each the summary estimate and its precision (e.g. confidence/credible interval) and measures of statistical heterogeneity. If comparing groups, describe the direction of the effect. | 14-16 |
|  | 20c | Present results of all investigations of possible causes of heterogeneity among study results. | 14-16 |
|  | 20d | Present results of all sensitivity analyses conducted to assess the robustness of the synthesized results. | 14-16 |
| Reporting biases | 21 | Present assessments of risk of bias due to missing results (arising from reporting biases) for each synthesis assessed. | 14-16 |
| Certainty of evidence | 22 | Present assessments of certainty (or confidence) in the body of evidence for each outcome assessed. | 14-16 |
| **DISCUSSION** | | |  |
| Discussion | 23a | Provide a general interpretation of the results in the context of other evidence. | 16-19 |
|  | 23b | Discuss any limitations of the evidence included in the review. | 18-19 |
|  | 23c | Discuss any limitations of the review processes used. | 18-19 |
|  | 23d | Discuss implications of the results for practice, policy, and future research. | 16-19 |
| **OTHER INFORMATION** | | |  |
| Registration and protocol | 24a | Provide registration information for the review, including register name and registration number, or state that the review was not registered. | 3 |
|  | 24b | Indicate where the review protocol can be accessed, or state that a protocol was not prepared. | 3 |
|  | 24c | Describe and explain any amendments to information provided at registration or in the protocol. | NA |
| Support | 25 | Describe sources of financial or non-financial support for the review, and the role of the funders or sponsors in the review. | NA |
| Competing interests | 26 | Declare any competing interests of review authors. | 19 |
| Availability of data, code and other materials | 27 | Report which of the following are publicly available and where they can be found: template data collection forms; data extracted from included studies; data used for all analyses; analytic code; any other materials used in the review. | NA |

***Table S3.*** *List of excluded studies and reasons for exclusion*

| **N.** | **Author (Year)** | **Reasons for exclusion** |
| --- | --- | --- |
| 1 | Bergmame et al., (2021) | The study is a scoping review. |
| 2 | Clarke et al., (2015) | The study is a research protocol. |
| 3 | Grey et al., (2013) | The study was a multisite clinical trial: participants were randomized to one of two Internet-based interventions. |
| 4 | Herbert et al. (2013) | The study is a systematic review |
| 5 | Huang et al., (2014) | The study includes adolescents with various chronic diseases (ACD). It do not focus exclusively on adolescents with type 1 or type 2 diabetes. HbA1c data are not reported. |
| 6 | Iafusco et al., (2011) | The study is not an RCT: participants were not randomly assigned to intervention and control groups. |
| 7 | Ibrahim et al., (2021) | No mean scores are reported. |
| 8 | Jaser et al., (2014) | The study is not a RCT: it compares two Internet-based intervention groups. |
| 9 | Losiouk et al., (2018) | HbA1c data are not reported. The questionnaires were administered to parents. The study is not a RCT. |
| 10 | Lunkenheimer et al., (2020) | The study does not focus exclusively on adolescents with type 1 or type 2 diabetes; it also includes adolescents with other chronic diseases. HbA1c data are not reported. |
| 11 | Nkhoma et al., (2021) | The study is a systematic review and meta-analysis. |
| 12 | Whittemore et al., (2012) | The study compares two Internet-based intervention groups. |
| 13 | Whittemore et al., (2016) | The study compares two Internet-based intervention groups. |

Bergmame, L., & Shaw, S. (2021). Clinical utility of psychoeducational interventions for youth with type 1 diabetes: a scoping review. *Continuity in Education*, *2*(1), 76.

Clarke, J., Vatiliotis, V., Verge, C. F., Holmes-Walker, J., Campbell, L. V., Wilhelm, K., & Proudfoot, J. (2015). A mobile phone and web-based intervention for improving mental well-being in young people with type 1 diabetes: design of a randomized controlled trial. *JMIR Research Protocols*, *4*(2), e4032.

Grey, M., Whittemore, R., Jeon, S., Murphy, K., Faulkner, M. S., Delamater, A., & TeenCope Study Group. (2013). Internet psycho-education programs improve outcomes in youth with type 1 diabetes. *Diabetes care*, *36*(9), 2475-2482.

Herbert, L., Owen, V., Pascarella, L., & Streisand, R. (2013). Text message interventions for children and adolescents with type 1 diabetes: a systematic review. *Diabetes technology & therapeutics*, *15*(5), 362-370.

Huang, J. S., Terrones, L., Tompane, T., Dillon, L., Pian, M., Gottschalk, M., ... & Bartholomew, L. K. (2014). Preparing adolescents with chronic disease for transition to adult care: a technology program. *Pediatrics*, *133*(6), e1639-e1646.

Iafusco, D., Galderisi, A., Nocerino, I., Cocca, A., Zuccotti, G., Prisco, F., & Scaramuzza, A. (2011). Chat line for adolescents with type 1 diabetes: a useful tool to improve coping with diabetes: a 2-year follow-up study. *Diabetes Technology & Therapeutics*, *13*(5), 551-555.

Ibrahim, N., Treluyer, J. M., Briand, N., Godot, C., Polak, M., & Beltrand, J. (2021). Text message reminders for adolescents with poorly controlled type 1 diabetes: A randomized controlled trial. *Plos one*, *16*(3), e0248549.

Jaser, S. S., Whittemore, R., Chao, A., Jeon, S., Faulkner, M. S., & Grey, M. (2014). Mediators of 12-month outcomes of two internet interventions for youth with type 1 diabetes. *Journal of pediatric psychology*, *39*(3), 306-315.

Losiouk, E., Lanzola, G., Del Favero, S., Boscari, F., Messori, M., Rabbone, I., ... & Quaglini, S. (2018). Parental evaluation of a telemonitoring service for children with type 1 diabetes. *Journal of telemedicine and telecare*, *24*(3), 230-237.

Lunkenheimer, F., Domhardt, M., Geirhos, A., Kilian, R., Mueller-Stierlin, A. S., Holl, R. W., ... & COACH consortium. (2020). Effectiveness and cost-effectiveness of guided internet-and mobile-based CBT for adolescents and young adults with chronic somatic conditions and comorbid depression and anxiety symptoms (youthCOACH CD): study protocol for a multicentre randomized controlled trial. *Trials*, *21*, 1-15.

Nkhoma, D. E., Soko, C. J., Bowrin, P., Manga, Y. B., Greenfield, D., Househ, M., ... & Iqbal, U. (2021). Digital interventions self-management education for type 1 and 2 diabetes: A systematic review and meta-analysis. *Computer methods and programs in biomedicine*, *210*, 106370.

Whittemore, R., Jaser, S. S., Jeon, S., Liberti, L., Delamater, A., Murphy, K., ... & Grey, M. (2012). An internet coping skills training program for youth with type 1 diabetes: six-month outcomes. *Nursing research*, *61*(6), 395-404.

Whittemore, R., Liberti, L. S., Jeon, S., Chao, A., Minges, K. E., Murphy, K., & Grey, M. (2016). Efficacy and implementation of an Internet psychoeducational program for teens with type 1 diabetes. *Pediatric Diabetes*, *17*(8), 567-575.

***Figure S4.*** *Insulin Usage (N = 594)*


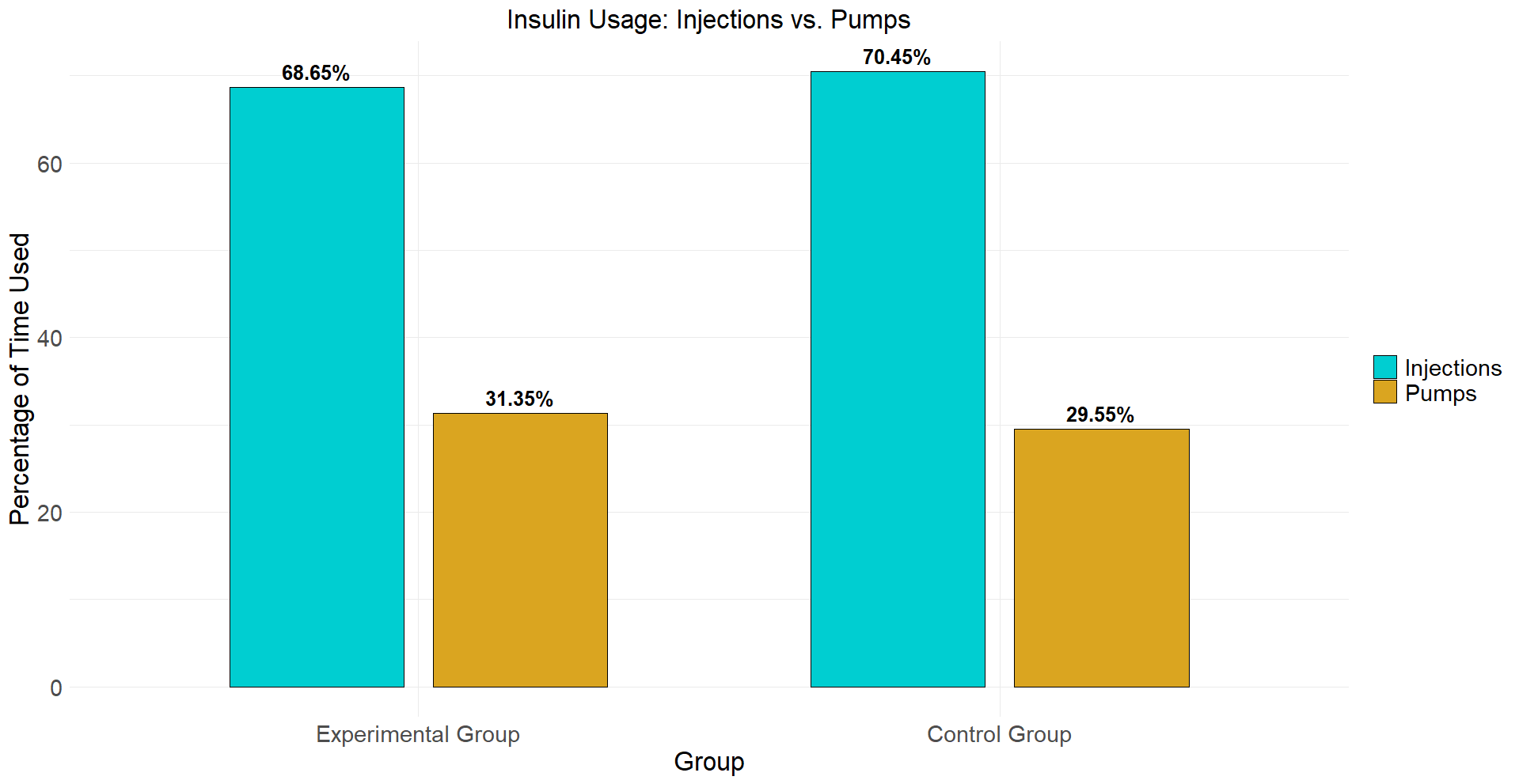


***Figure S5.*** *Comparison of baseline means HbA1c (%) (N = 574) and BMI levels (N = 313) between experimental vs. control groups*


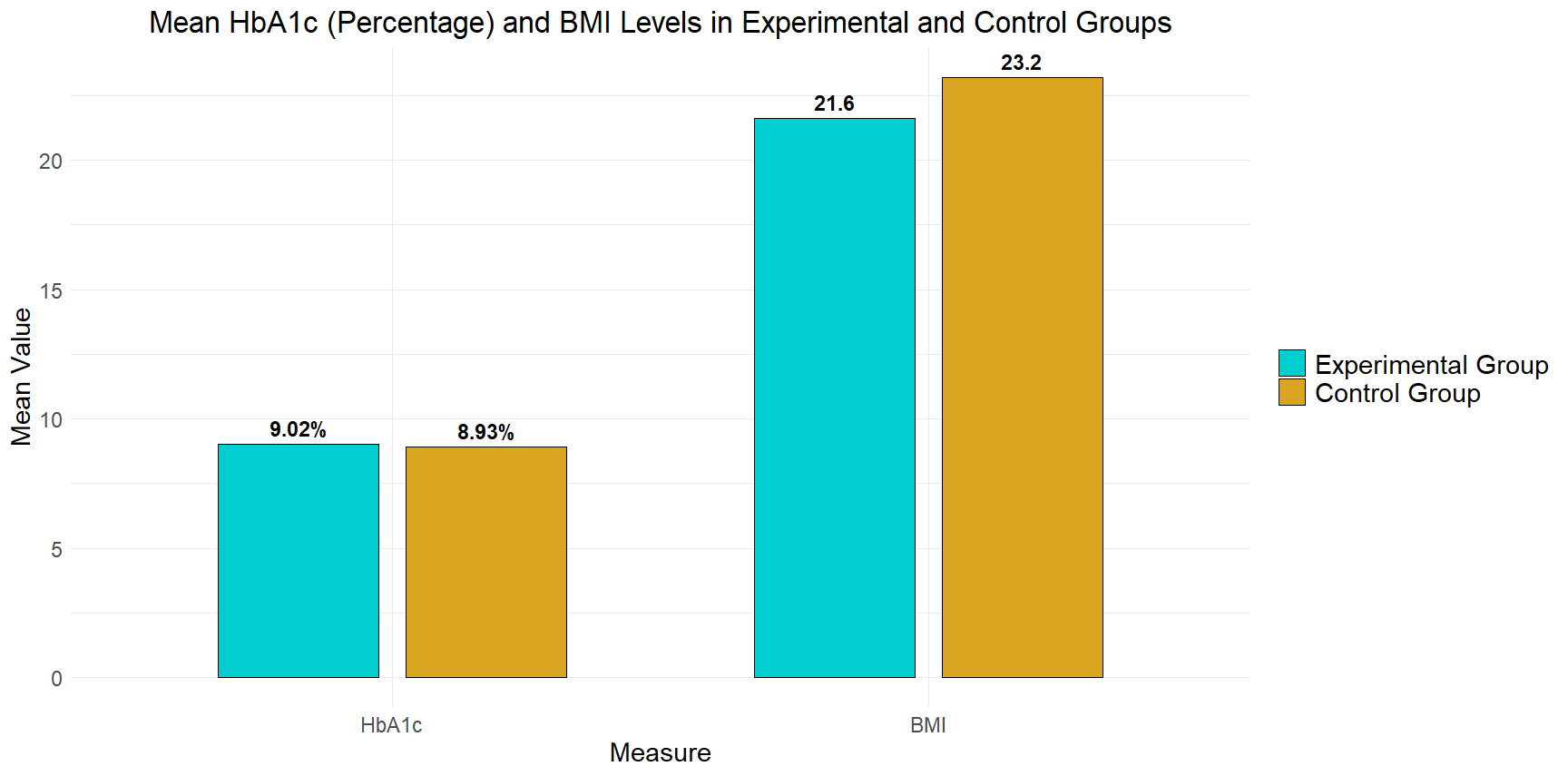


***Figure S6.*** *Forest plot of effect sizes for impact of diabetes subscale of DQOLY (k = 4)*


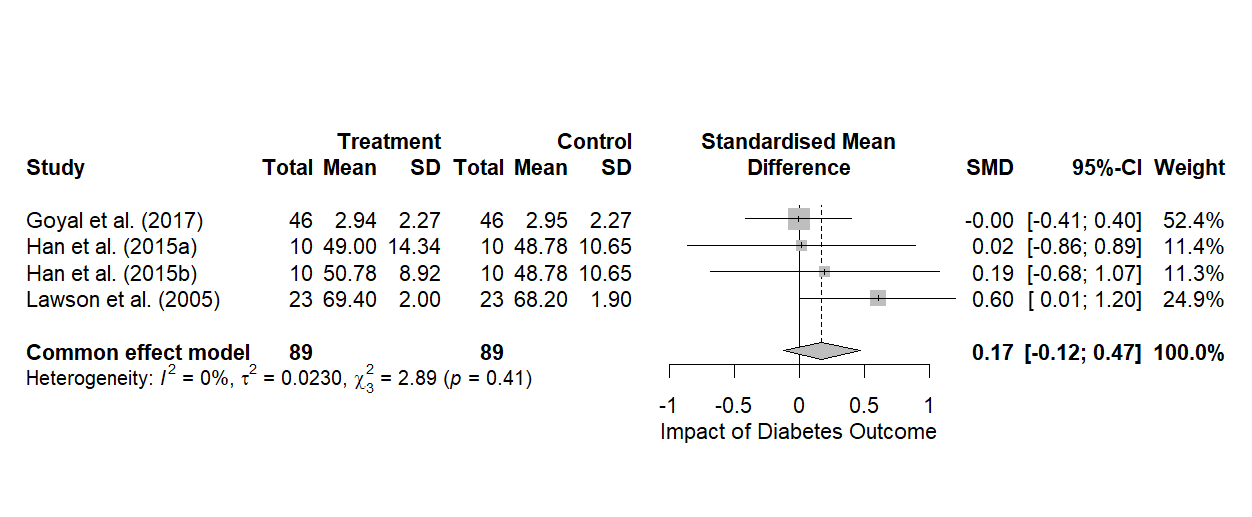


***Figure S7.*** *Forest plot of effect sizes for worries about diabetes subscale of DQOLY (k = 4)*

*
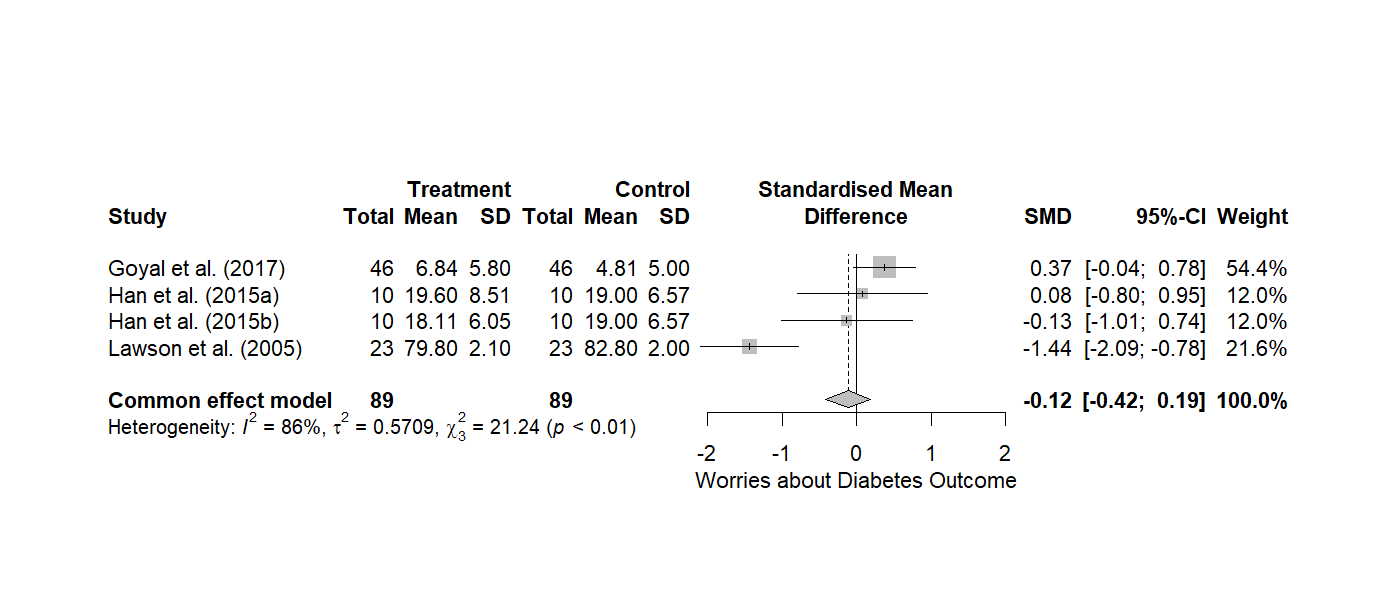
*

***Figure S8.*** *Funnel plots assessing publication bias for impact of diabetes*

*
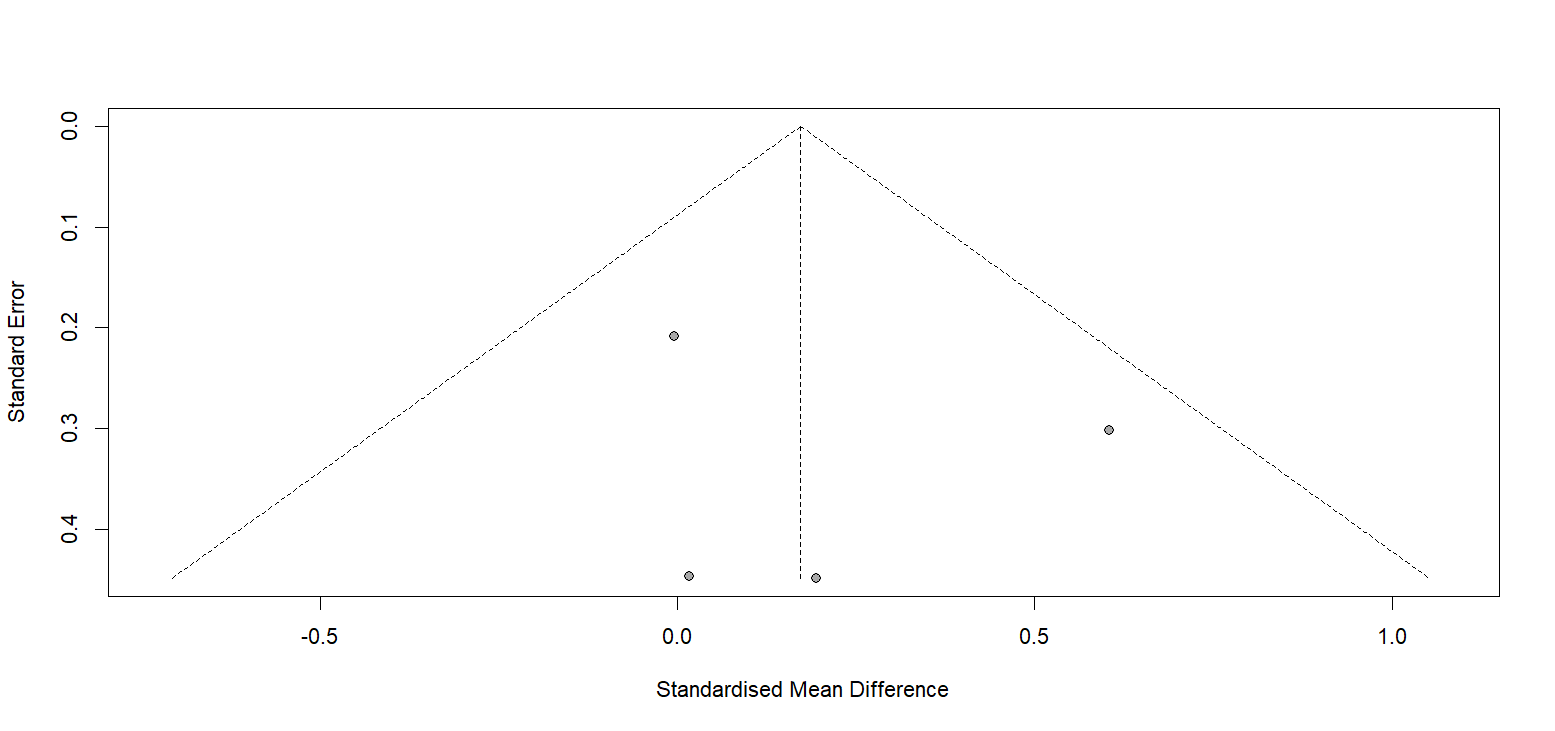
*

***Figure S9.*** *Funnel plots assessing publication bias for worries about diabetes*

*
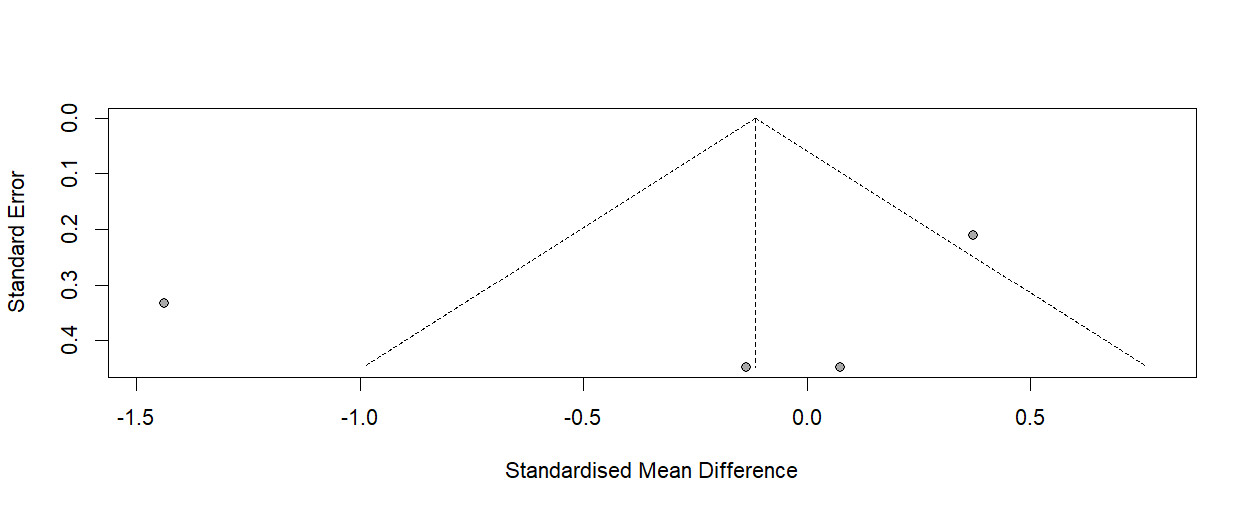
*
